# Editorial: Intra- and Inter-individual Variability of Executive Functions: Determinant and Modulating Factors in Healthy and Pathological Conditions

**DOI:** 10.3389/fpsyg.2019.00432

**Published:** 2019-03-08

**Authors:** Sarah E. MacPherson, Celine R. Gillebert, Gail A. Robinson, Antonino Vallesi

**Affiliations:** ^1^Human Cognitive Neuroscience, Department of Psychology, University of Edinburgh, Edinburgh, United Kingdom; ^2^Centre for Cognitive Ageing and Cognitive Epidemiology, University of Edinburgh, Edinburgh, United Kingdom; ^3^Department of Brain and Cognition, KU Leuven, Leuven, Belgium; ^4^Department of Experimental Psychology, University of Oxford, Oxford, United Kingdom; ^5^Neuropsychology Research Unit, School of Psychology, The University of Queensland, Brisbane, QLD, Australia; ^6^Queensland Brain Institute, The University of Queensland, Brisbane, QLD, Australia; ^7^Department of Neuroscience & Padova Neuroscience Center, University of Padua, Padua, Italy; ^8^Brain Imaging and Neural Dynamics Research Group, IRCCS San Camillo Hospital, Venice, Italy

**Keywords:** executive abilities/function, cognitive aging, intelligence, expertise, bilingualism, development, inter-individual and intra-individual differences, cognitive reserve

Executive functioning generally refers to the ability to organize thought and action based on intentions and goals, especially in novel, complex or difficult situations. Executive functioning is a multifaceted psychological construct that may be depicted as a set of related but separable high-level cognitive abilities, possibly supported by the prefrontal cortex and implemented by larger brain networks (Shallice and Burgess, 1996; Miyake et al., 2000) but see Duncan et al. (1997). Many models exist that emphasize commonalities or differences among various executive functions (EF). While the number and type of EF that exist remain a topic of debate, most authors would agree that EF show high intra- and inter-individual variability in terms of their cognitive and behavioral manifestations.

But what are the determinant and modulating factors that might explain the variability across EF? Do neuro-anatomical or neuro-functional factors and/or the environment influence EF? The overall goal of our research topic was to provide a forum to explore the contributions of different research groups investigating intra- and inter-individual variability in EF. We welcomed empirical, theoretical and meta-analytical work involving both clinical and healthy human populations. We were impressed by the number of authors who did indeed rally to our call; our research topic resulted in contributions from 187 authors and 39 published articles. At the time of writing, our research topic has resulted in an impressive 62,809 total views and 5,728 article downloads. We hope after reading these articles, you will be more sensitive to the various factors that contribute to intra- and inter-subject variability in EF and will be inspired to consider these when studying EF in both healthy and pathological conditions.

What follows is a brief overview of the contributions to our research topic. We aim to highlight some of the key influences on EF variability, and some of the interesting questions to emerge from these articles that we hope will encourage and influence future research. We appreciate that this editorial cannot fully do our research topic justice in terms of the breadth and depth of topics/questions included and so we encourage you to read further the contributions that these articles offer to the research area of EF.

## Developmental Trajectory of EF

Although EF are thought to be multifaceted, the general consensus in the developmental literature is that there is a unitary EF factor in preschool children (Wiebe et al., 2008). This develops into a two factor model (working memory and inhibition/shifting) in primary school-aged children (Brydges et al., 2014) and finally, manifests into a three-factor model in adolescence (Latzman and Markon, 2010). By late adulthood, EF become more unidimensional again (sometimes referred to as the differentiation-dedifferentiation hypothesis, Wiebe et al., 2011; Brydges et al., 2012).

Developing this work further, several contributions in our research topic examine the tripartite model in children, adolescents and young adults. Messer et al. examined the relationship between 10 verbal and non-verbal EF tasks in 128 typically developing primary-school aged children. Their aim was to determine how performance on these distinct EF tasks relates with one another. The exploratory factor analysis produced two factors, one inhibition factor containing the two inhibition tasks, and a general EF factor that included the other shifting, working memory/updating, fluency, and planning tasks. Here, the findings of a two-factor EF model in primary-school aged children was replicated, although the nature of the factors varied. It may be that different factor structures are the product of task impurity (Miyake et al., 2000) where distinct tasks tapping the same EF function have different relationships with other EF tasks. The selection of the EF components considered is often task-based but Messer et al. propose that future work should select EF tasks based on evidence from brain/behavior relationships.

Developmental changes in the factor structure of EF factors are thought to be related to maturation in the prefrontal cortex, a region which continues to experience considerable changes in adolescence (Yakovlev and Lecours, 1967). Neuroimaging studies have shown a linear increase in prefrontal white matter volume due to increased myelination during adolescence (Barnea-Goraly et al., 2005). There is also a reduction in gray matter volume (Gogtay et al., 2004) due to a reduction in synaptic density but an increase in the remaining synapse's efficiency (Blakemore and Choudhury, 2006). This brain development may continue in late adolescence and early 20s (Gogtay et al., 2004) and not reach stability until around 30 years of age (Sowell et al., 2003). In our research topic, Smolker et al. examined whether individual differences in gray and white matter measures are associated with individual differences in EF in young adults in their 20s. They administered 6 tasks tapping the three constructs of the tripartite model to 251 adults. Smolker et al. reported a common factor influencing performance on all EF tasks, as well as updating-specific and shifting-specific factors. In terms of associations between the EF and neuroanatomical measures, they found the common EF was related to several gray matter and fractional anisotropy characteristics. The updating-specific factor was associated with gray matter characteristics only, whereas the shifting-specific factor was associated with several white matter properties (see Smolker et al.). In another study involving the same cohort, Reineberg et al. examined the relationship between fMRI resting state network connectivity and individual differences in separable components of EF. The authors found that individuals with higher performance on the shifting-specific factor had more positive connectivity between the frontoparietal and visual networks, whereas individuals with higher performance on the common EF factor exhibited increased connectivity between sensory and default mode networks. These results uncover more specific relationships between connectivity and EF.

Contributors to our research topic have also examined the latent factor structure of EF in relation to neurodevelopmental conditions such as autism (Filipe et al.) and dyslexia (Doyle et al.).  Filipe et al. highlighted an important bidirectional link between EF skills (divided attention, working memory, set-switching, inhibition) and prosodic abilities, although children with high functioning autism and controls did not differ. Doyle et al. examined how different EF contribute to reading ability by studying children with dyslexia and age-matched controls. Proficient reading is thought to require EF to switch between multiple reading processes, inhibit irrelevant information, and hold and update speech. However, the exact profile of spared and impaired EF associated with dyslexia remains unclear with some studies reporting EF impairments (Bexkens et al., 2014) and others not (Poljac et al., 2010). Doyle et al. found that the inhibition and updating composite scores significantly predicted reading ability and the likelihood of dyslexia whereas switching did not. These findings encourage future work to explore EF training as an intervention for children with dyslexia, which in turn, might transfer to improved reading ability.

## Aging and EF

Moving to the other end of the spectrum and the influence of cognitive aging on EF, studies consistently report that healthy older adults perform poorer than younger adults on EF tasks (see MacPherson and Della Sala, 2015). Frontal lobe theories of cognitive aging propose that the age-related decline on EF tasks is either due to overall frontal lobe decline (West, 1996) or more specific dorsolateral prefrontal decline (MacPherson et al., 2002). In support of these theories, neuroimaging studies have demonstrated that the frontal lobes are especially vulnerable to age-related changes in terms of overall cortical volume, cortical thickness, and white matter compared to other brain regions (Fjell et al., 2009).

While there seems little doubt that healthy and pathological aging result in structural and functional changes in the frontal lobes and poorer EF performance (Cabeza and Dennis, 2013), it remains less clear whether older adults experience similar patterns of deterioration across different EF. In the cognitive aging literature, most attention has been placed on examining intra-individual variability across task trials (Dykiert et al., 2012), and less attention has been placed on “dispersion”—the study of variability across cognitive tasks (Hilborn et al., 2009). Some cross-sectional and longitudinal aging studies have reported that dispersion reduces with age (Rabbitt et al., 2004) but others have found an increase in dispersion with age (Sosnoff and Newell, 2006). In our research topic, Buczylowska and Petermann examined a large group of 444 healthy adults aged from 18 to 99 years performing the NAB Executive Functions Module, which includes subtests of planning, mazes, letter fluency, judgment, categories, and word generation. The authors found that the variability across EF tasks decreased with age and there were increasing intercorrelations between tasks. These findings suggest EF in late adulthood become unidimensional in nature and provide support for the dedifferentiation hypothesis.

On a different note, our research topic also includes work further examining the relationship between EF performance and neurodegenerative changes in older adults. For example, Di Tella et al. explored the relationship between EF, specifically selection, and changes in cortical thickness in the inferior regions of the frontal lobes in patients with Parkinson's disease (PD) with predominantly left or right cortical involvement. Twenty-one PD patients and 19 controls performed a noun-verb generation task and a second verb-noun derivation task. Only PD patients with left-sided but not right-sided atrophy were impaired compared to the controls on both linguistic tasks. Furthermore, in the left-sided PD patients, significant correlations between accuracy and RTs and cortical thickness in the left inferior frontal gyrus (IFG) were found. Di Tella et al. conclude that linguistic and EF processes interact in the left IFG during word production tasks involving selection and suggest that future work should consider these structural cortical asymmetries in PD further.

In another study, Palermo et al. examined PD patients' partial or complete unawareness of their involuntary movements (i.e., dyskinesias-reduced-self-awareness, DRSA) in relation to performance on response-inhibition tasks and hypofunctionality in the anterior cingulate cortex (ACC). Previously, Maier et al. (2016) demonstrated that impaired self-awareness in PD patients was related to reduced metabolism in the bilateral frontal regions including the medial frontal gyrus (particularly the ACC), which has been associated with impaired self-awareness in Alzheimer's disease (AD; Amanzio et al., 2011), acquired brain injury (Palermo et al., 2014), bipolar disorder (Palermo et al., 2015), and schizophrenia (Orfei et al., 2010). Palermo et al. extend their own work to 27 PD patients presenting with motor fluctuations and dyskinesias who underwent event-related functional MRI while performing a response-inhibition GO/No-GO task. They found that reduced bilateral ACC involvement, as well as in the bilateral anterior insular cortex and right dorsolateral prefrontal cortex, was related to the presence of DRSA. Furthermore, DRSA scores significantly correlated with percent errors on the No-GO condition. The authors conclude that the reduction in self-awareness of dyskinesias in PD may be due to a specific impairment in EF related to metacognitive awareness.

## Environmental Influences on EF

Certain lifetime experiences have been proposed to “protect” against the impact of brain damage, which may account for the variability in cognitive performance that can be found in patients with similar degrees of brain pathology. These protective influences have been referred to as cognitive reserve (CR; Stern, 2002). As CR cannot be assessed directly, a number of indicators have been proposed as CR proxies. Education level is a commonly adopted index of CR, as is literacy attainment, which is typically measured using single word reading tests such as the National Adult Reading Test (NART; Nelson and Willison, 1991). CR has predominantly been investigated in relation to neurodegenerative disorders such as AD, traumatic brain injury and healthy aging (Harrison et al., 2015), where individuals who have higher levels of education and/or NART IQ are found to have less cognitive impairment than individuals with lower levels of education and/or NART IQ (e.g., Singh-Manoux et al., 2011).

Readers of this research topic will be most keen to consider the influence, if any, of CR on EF. Indeed, there is some evidence to suggest that EF are susceptible to the mitigating effects of CR. Educational attainment has been found to predict EF performance both in healthy aging (Meguro et al., 2001) and AD (Scarmeas et al., 2006). Higher education in stroke patients has also been associated with better performance on EF tests (Ojala-Oksala et al., 2012). More recently, MacPherson et al. (2017) retrospectively examined patients with frontal lesions and found that NART IQ (and age) predicted performance on EF tests (i.e., Stroop Test and letter fluency). Therefore, there do appear to be protective effects of CR on EF and this may explain some of the inter-individual variability in performance on certain tasks across patients with similar levels of brain pathology.

In the current research topic, De Felice and Holland studied whether CR factors might have differential effects on individuals' performance on distinct EF tasks (i.e., fluency, Trail-Making Test, and digit span forwards and backwards) depending upon their age. They compared younger (22–31 years), middle-old (59–71 years), and old-old (76–91 years) groups. They reported a trend that old-old adults had the greatest dispersion index, and this was coupled with poorer task performance compared to the younger and middle-old groups. The authors conclude that middle-old adults with better cognition exclusively benefit from higher CR and demonstrate a dispersion index equivalent to younger adults.

Both education and NART IQ have been criticized as indices of CR (Jones et al., 2011). Education varies in the quality, availability and subjects taught across different countries and social groups whereas dyslexia and other learning difficulties are detrimental to performance on literacy attainment and can result in inaccurate estimates (Ikanga et al., 2016). Moreover, other real-life factors that may modify cognitive decline such as occupational attainment (Garibotto et al., 2008) and leisure activities (Wilson et al., 2002) are considered less by researchers. Given that different indices might contribute to CR, Nucci et al. (2012) devised the Cognitive Reserve Index questionnaire (CRIq), which provides a measure of overall cognitive reserve but also distinct dimensions that contribute to the overall score (i.e., education, occupational attainment, and leisure time). In our research topic, Moretti et al. considered the potential role of distinct CR factors and general slowing on modulating cognitive flexibility in young, middle-age and older adults. Using the CRIq, the authors report that education was the only index associated with reduced switch costs under time pressure and highlight the importance of using tools designed to distinguish between different CR dimensions to understand better which life-long experiences protect different cognitive functions (Puccioni and Vallesi, 2012a,b).

Another potential life course factor thought to play a protective role against cognitive decline is bilingualism. Bilingualism is a hot topic in the EF literature given that some work has shown that bilingualism results in improved cognitive function in healthy aging (Bak et al., 2014) and post-stroke (Alladi et al., 2016) and is associated with a delay in the onset of mild cognitive impairment (Ramakrishnan et al., 2017), dementia (Bialystok et al., 2007) and behavioral variant frontotemporal dementia (Alladi et al., 2017). While there is considerable debate around the presence, magnitude and mechanisms associated with the bilingualism effect (Freedman et al., 2014), some research has shown positive effects on EF associated with speaking more than one language (Bak, 2016).

When studying bilingualism, it is important to know whether such benefits are specific to language or are domain-general. While some propose that long-standing bilingualism affects non-linguistic executive control, as smaller switch costs are reported in bilinguals performing non-linguistic tasks compared to monolinguals (Prior and Macwhinney, 2010), others have not found a bilingual advantage (Paap et al., 2017). In this research topic, Timmer et al. argue that the currently used linguistic and non-linguistic control measures in bilinguals may not be reliable. Using linguistic and non-linguistic switch tasks administered to Catalan-Spanish-English trilinguals, they demonstrated that the cost of switching between languages/tasks compared to repeating the same language/task is a reliable measure of cross-talk between linguistic and non-linguistic executive control and that there are at least some shared processes across the tasks. Timmer et al.'s work makes us reconsider the reliability of the measures used to study bilingualism. Perhaps bilingualism can result in domain-general benefits but, for now, the jury is still out.

While the bilingualism debate will continue for some time, our research topic also includes studies examining whether expertise for other skills, such as playing strategy board games, goes beyond the specific skill itself and results in a more general advantage for cognitive skills. Training in board games such as chess may potentially enhance an individual's working memory abilities (WM) as players need to hold in WM several potential offensive moves and their opponent's predicted responses to each of those future moves. Consistently, however, experimental studies involving chess experts and novices performing WM tasks using chessboards and faces or scenes have reported group differences between the experts and novices for chessboard stimuli but not other stimulus types (Bartlett et al., 2013). The neuroimaging results are less consistent with some studies reporting an increase in activation in the fusiform gyrus in experts compared to novices in response to chessboards (Bilalić et al., 2011), yet others report no differences (Krawczyk et al., 2011).

In our research topic, Jung et al. examined whether expertise for the Korean strategy board game, Baduk, goes beyond the game itself and how it maps on networks associated with cognitive abilities that are not directly trained. The authors adopted a data-driven, whole-brain multivariate analytic approach as part of a connectome-wise association study (CWAS) to examine brain-behavior relationships in experts. Seventeen Baduk experts performed a visual n-back WM task including both face matching and spatial location matching conditions. They found that experts did not show an increase in WM ability compared to novices suggesting that expertise does not transfer to other cognitive abilities. However, experts did have greater activation in the superior parietal cortex during the face WM task and greater connectivity between frontal and parietal regions and between frontal and temporal regions. These findings provide evidence that experts undergo reorganization of functional interactions between brain regions associated with WM., showing that experience-related brain changes may be more sensitive than behavioral ones.

In another study of expertise, Visalli and Vallesi examined the expertise of quality-control employees, focusing on whether visual search expertise extends to generalized search behaviors. In particular, they focused on monitoring processes, the goal of which is to “quality check” in order to enhance behavior (see Vallesi, 2012 for an overview). Twenty-four fruit quality controllers and 23 controls performed a computerized visual search task with one block containing oranges (expert knowledge) and one block containing the Smurfette doll (neutral knowledge). They found that quality-controllers were significantly faster than controls in the conditions thought to require monitoring processes (i.e., all target-present and target-absent conditions except the orange-present condition). These results suggest that top-down processes in visual search can be enhanced through immersive real-life experience beyond visual expertise advantages. Therefore, the findings of associations between expertise and improved EF are not consistent and may depend on the type of expertise and the tasks involved.

## Intelligence and EF

Some theories suggest that the frontal lobes play a role in general control processes that are employed when performing diverse cognitive tasks, regardless of the type of information being processed (e.g., Duncan, 2001; Miller and Cohen, 2001). Neuroimaging studies have demonstrated activation in the lateral frontal cortex, dorsomedial frontal cortex, and anterior insula, as well as the intraparietal sulcus, when performing difficult tasks across different domains (Fedorenko et al., 2012). The activation of these regions when performing distinct tasks has been referred to as the multiple-demand (MD) network, and this network is thought to be central in the organization of several types of behavior (Duncan, 2005).

The activity in the MD network when performing different cognitive tests has been associated with fluid intelligence (e.g., Woolgar et al., 2010) and this has led researchers to investigate the relationship between fluid intelligence and EF. Research studies have found that fluid intelligence positively correlates with EF measures and frontal lobe lesions impair performance on tests of fluid intelligence (Duncan et al., 1995), particularly lesions involving MD regions (Woolgar et al., 2010). Furthermore, activation in the MD network is found when individuals perform fluid intelligence tests (Duncan et al., 2000). Interestingly, increasing complexity in nonverbal reasoning tasks has recently been associated with abnormal MD network activation in individuals with developmental corpus callosal dysgenesis (Hearne et al., 2018). These findings suggest that it may be a decline in fluid intelligence which underlies the EF impairments reported in frontal patients. Roca et al. (2010) demonstrated that impaired performance in frontal patients on EF tests such as the Wisconsin Card Sorting Test, verbal fluency and the Iowa Gambling Task can be explained by fluid intelligence impairments, although Robinson et al. (2012) showed the opposite for verbal fluency. However, for other EF tasks such as the Hayling Sentence Completion test and the Stroop test, frontal patients' impairments could not be accounted for by reduced fluid abilities (Roca et al., 2010; Cipolotti et al., 2016: for a similar finding in schizophrenia see Martin et al., 2015). Moreover, although Barbey et al. (2012) identified shared neural substrates in the frontal and parietal cortex for EF and general intelligence (*g*), there were additional brain regions specific to EF (e.g., the left anterior pole) and brain regions specific to *g* (e.g., the left inferior occipital gyrus and the right superior and inferior parietal lobe).

In our research topic, contributors have further examined the relationship between EF abilities and intelligence in healthy and patient populations. Nęcka et al. investigated whether self-control (SC) is subserved by EF in 296 healthy younger volunteers through the administration of 5 EF tasks, 3 self-report SC measures and two fluid intelligence tests. Using a structural equation modeling approach, three latent variables of executive control, behavioral control, and fluid intelligence (Gf) were extracted. Surprisingly, Nęcka et al. did not find any EF-SC or Gf-SC relationships. However, a reasonably strong EF-Gf relationship was found. The authors conclude that SC may not depend on the strength of executive control, at least in healthy adults.

Moving onto studies involving frontal patients, Chan et al. examined whether the memory impairments often reported in frontal patients are better explained by declines in fluid intelligence or EF. Thirty-nine patients with focal frontal lesions were assessed on tests of recall and recognition memory, fluid intelligence, and EF. As in their previous work (e.g., MacPherson et al., 2016), Chan et al. found that their frontal patients were impaired on both recall and recognition memory tests compared to healthy controls. Importantly, however, whereas fluid intelligence was the strongest predictor of recall deficits, recognition memory was not related to intelligence or EF performance. Overall, Chan et al. show that the nature of the frontal deficit on different memory tasks may be separable in relation to other clinical and cognitive factors influencing performance.

This has been a very brief overview of the contents of our research topic. While our editorial cannot encompass all author contributions, it highlights some of the interesting findings that can be found within the topic, with the hope of encouraging you to read further. And of course, not all determining and modulating factors that contribute to EF variability have been discussed. When we proposed this research topic to Frontiers, our aim was to provide a forum where the contributions of different research groups investigating intra- and inter-individual variability in EF could be discussed. We hope you find this forum a valuable contribution to the EF literature and it generates as many questions as it answers.

## Author Contributions

SM wrote the first draft of the manuscript. All authors listed have made a substantial, direct and intellectual contribution to the work, and approved it for publication.

### Conflict of Interest Statement

The authors declare that the research was conducted in the absence of any commercial or financial relationships that could be construed as a potential conflict of interest.
